# Incidence or Coincidence: Subaortic Membrane, Bicuspid Aortic Valve, and Aortic Root Dilatation in a Patient Presenting With Stroke

**DOI:** 10.7759/cureus.105363

**Published:** 2026-03-17

**Authors:** Goitom Weldearegay, Sofia R Khan, Francesca Cali, Asher Gorantla, Suzette Graham-Hill

**Affiliations:** 1 Internal Medicine, State University of New York Downstate Health Sciences University, Brooklyn, USA; 2 Cardiology, State University of New York Downstate Health Sciences University, Brooklyn, USA; 3 Cardiology, Kings County Hospital, Brooklyn, USA

**Keywords:** aortic root dilation, atherosclerotic plaque, bicuspid aortic valve disease, central retinal artery occlusion (crao), subaortic valvular membrane

## Abstract

Bicuspid aortic valve (BAV) is the most common congenital cardiac malformation and is frequently associated with progressive aortopathy. Subaortic membrane, a discrete form of left ventricular outflow tract (LVOT) obstruction, is uncommon in adults and rarely coexists with BAV outside of complex congenital syndromes. The simultaneous presence of BAV, aortic root dilation, and a subaortic membrane represents an unusual anatomical constellation with important diagnostic and clinical implications.

We present the case of a 49-year-old man with no known medical history who developed sudden, painless monocular vision loss and was diagnosed with left central retinal artery occlusion (CRAO). Neurovascular imaging revealed mild carotid atherosclerosis and incidental ascending aortic dilation. Transthoracic echocardiography (TTE) demonstrated a probable BAV, left atrial enlargement, and a suspected subaortic membrane with elevated LVOT gradients. Transesophageal echocardiography confirmed BAV morphology, mild aortic stenosis, and aortic root dilation measuring 4.2 cm, along with severe atherosclerotic plaque in the aortic arch, suggesting an embolic source.

This case highlights the importance of comprehensive cardiac evaluation in patients with unexplained embolic events and expands the spectrum of structural abnormalities associated with BAV. Recognition of this rare triad is important for appropriate surveillance, risk stratification, and multidisciplinary management.

## Introduction

Bicuspid aortic valve (BAV) affects approximately 1%-2% of the population and is strongly associated with progressive aortopathy, including dilation of the aortic root and ascending aorta [1,2]. Patients with BAV have a significantly higher lifetime risk of aortic aneurysm formation, dissection, and valvular dysfunction compared with the general population [3]. Aortic root dilation represents a high-risk phenotype that influences surveillance intervals and surgical thresholds [2,4].

Subaortic membrane, a cause of discrete subaortic stenosis (DSS), is far less common in adults and is typically identified in childhood or in association with complex congenital heart disease [5]. Although rare, several reports describe the coexistence of BAV and subaortic membrane, suggesting a shared developmental or hemodynamic mechanism [6,7]. These lesions may contribute to progressive left ventricular outflow tract (LVOT) obstruction, accelerated valvular degeneration, and increased embolic risk.

The combination of BAV, aortic root dilation, and subaortic membrane is unusual and may mimic other congenital obstructive syndromes, though it does not meet the criteria for Shone complex. Accurate characterization requires multimodality imaging, particularly when transthoracic echocardiography (TTE) is limited. Aortic root dilation in the setting of BAV represents a distinct high‑risk phenotype that influences surveillance strategies and timing of surgical intervention [2,4]. Mechanistic studies suggest that aortic dilation in BAV reflects intrinsic abnormalities of aortic wall structure and altered flow-related shear stress rather than valvular dysfunction alone [5].

This case describes the identification of this rare triad during evaluation for central retinal artery occlusion (CRAO), underscoring the importance of comprehensive cardiovascular assessment in patients presenting with unexplained embolic events.

## Case presentation

A 49-year-old man with no known medical history and long-standing tobacco and marijuana use presented with sudden, painless monocular vision loss in the left eye. He reported going to bed in his usual state of health at 7:00 p.m. and awakening at 2:00 a.m. with complete loss of vision in the left eye. Believing the symptoms would resolve spontaneously, he returned to sleep. Upon awakening again at 8:00 a.m. with persistent visual loss, he sought emergency care. He denied headache, trauma, diplopia, weakness, numbness, dysarthria, or other neurologic symptoms.

On arrival, his blood pressure was markedly elevated at 168/119 mmHg. Neurologic examination revealed a relative afferent pupillary defect in the left eye and profound visual loss with only light perception. The remainder of the cranial nerve, motor, sensory, and cerebellar examinations were normal. His National Institute of Health (NIH) Stroke Scale score was 2 due to a visual field deficit. Ophthalmologic evaluation demonstrated a pale macula with a cherry-red spot, consistent with CRAO.

Initial laboratory studies were largely unremarkable aside from mild anemia. The basic metabolic panel, coagulation studies, and inflammatory markers showed no acute abnormalities. A broad hypercoagulability and autoimmune workup was initiated, given the patient’s age and lack of traditional vascular risk factors (Table 1).

**Table 1:** Key laboratory results ^*^Values slightly above or below the reference range. WBC: white blood cell count; Hgb: hemoglobin; Hct: hematocrit; Plt: platelet; MCV: mean corpuscular volume; CO₂: bicarbonate; BUN: blood urea nitrogen; GFR: glomerular filtration rate; AST: aspartate aminotransferase; ALT: alanine aminotransferase; PT: prothrombin time; INR: international normalized ratio; aPTT: activated partial thromboplastin time; POC: point‑of‑care; LDL: low‑density lipoprotein; HbA1c: hemoglobin A1c

Laboratory test	Result	Units	Reference range
Complete blood count			
WBC	6.83	K/µL	4.50-10.90
Hgb	13.4^*^	g/dL	14.0-18.0
Hct	40.1^*^	%	42-52
Plt count	253	K/µL	130-400
MCV	84.4	fL	78-95
Basic metabolic panel			
Sodium	140	mmol/L	135-145
Potassium	3.6	mmol/L	3.5-5.0
Chloride	108^*^	mmol/L	98-107
CO₂	22^*^	mmol/L	23-29
BUN	16	mg/dL	7-20
Creatinine	1.1	mg/dL	0.6-1.3
Calcium	9.4	mg/dL	8.5-10.5
Anion gap	10	-	8-16
Estimated GFR	82.3	mL/min/1.73m²	>60
Liver function tests			
AST	18	U/L	10-40
ALT	14	U/L	7-56
Alkaline phosphatase	106	U/L	44-147
Total bilirubin	0.3	mg/dL	0.1-1.2
Coagulation profile			
PT	11.7	sec	11-13.5
INR	1.1	-	0.8-1.2
aPTT	32	sec	25-35
Glucose			
Serum glucose	95	mg/dL	70-99 (fasting)
POC Glucose	91-101	mg/dL	70-140
Lipid profile			
LDL Cholesterol	96	mg/dL	100-129
Hemoglobin A1c			
HbA1c	5.5	%	4.0-5.6

Non-contrast CT of the head showed no acute intracranial pathology. CT angiography of the head and neck revealed no large-vessel occlusion or significant carotid stenosis but demonstrated mild atherosclerotic plaque in the right carotid siphon and incidental dilation of the ascending aorta. MRI of the brain showed chronic lacunar infarcts and nonspecific periventricular and callosal T2/fluid-attenuated inversion recovery (FLAIR) hyperintensities, raising consideration of demyelinating disease versus chronic microvascular ischemia. A lumbar puncture was performed, and cerebrospinal fluid analysis was normal, with no evidence of infection or inflammatory demyelinating disease.

**Figure 1:**
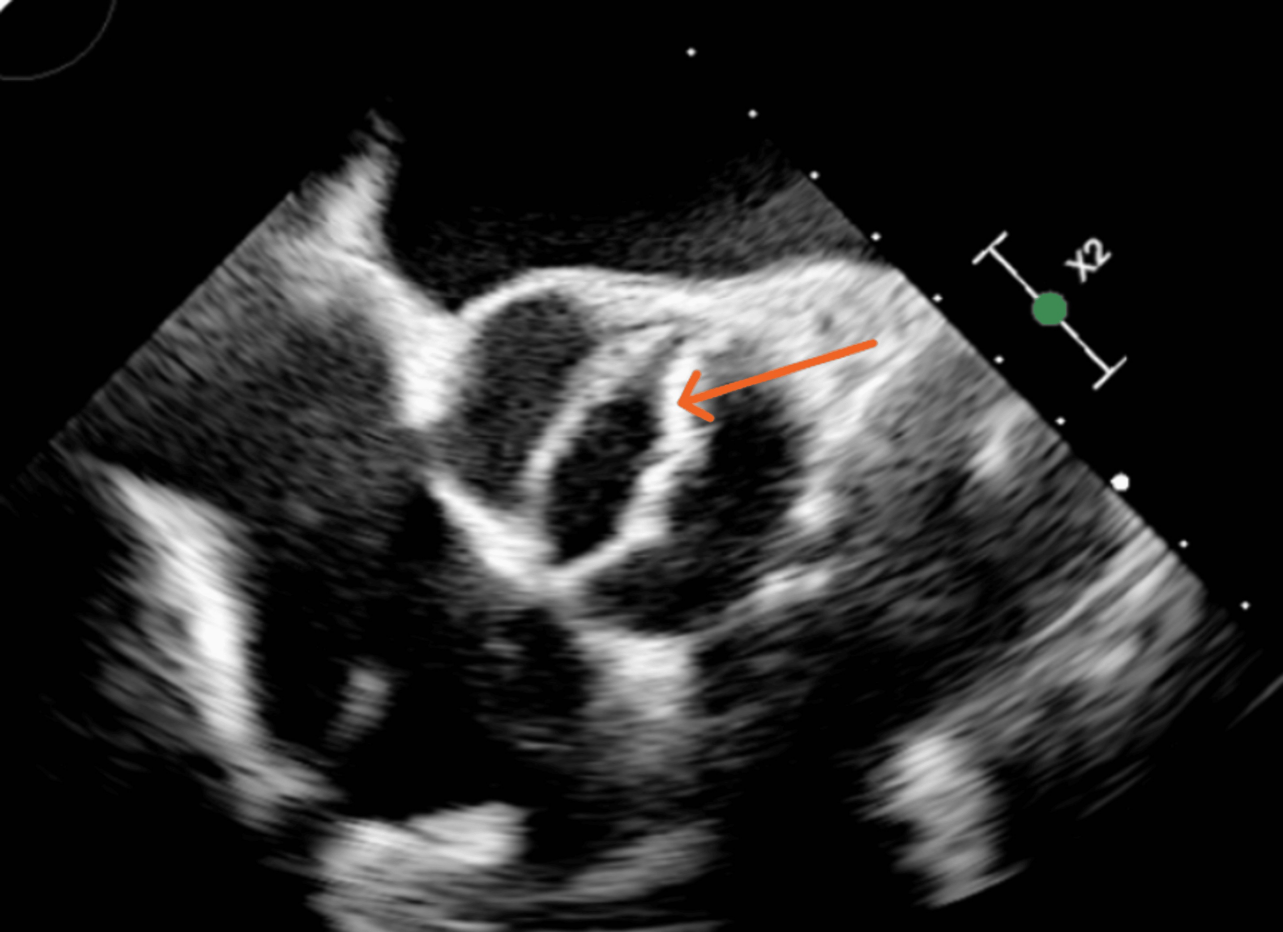
Parasternal short-axis TTE view demonstrating a BAV with fusion of the right and left coronary cusps (red arrow). The fused commissure produces the characteristic elliptical systolic opening pattern associated with bicuspid valve morphology X2 denotes a two-fold zoom applied by the echocardiography machine, indicating image magnification rather than an anatomical distance measurement. BAV: bicuspid aortic valve; TTE: transthoracic echocardiogram

**Figure 2:**
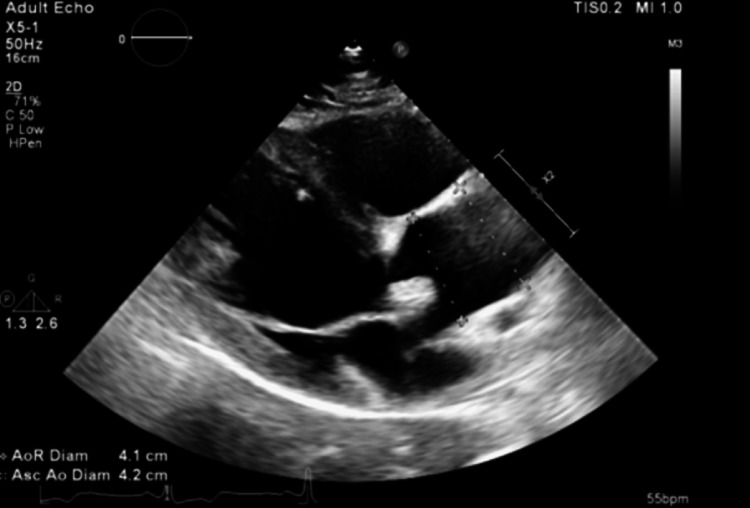
Dilation of the aortic root and proximal aorta depicted by the dotted lines X2 denotes a two-fold zoom applied by the echocardiography machine, indicating image magnification rather than an anatomical distance measurement.

**Figure 3:**
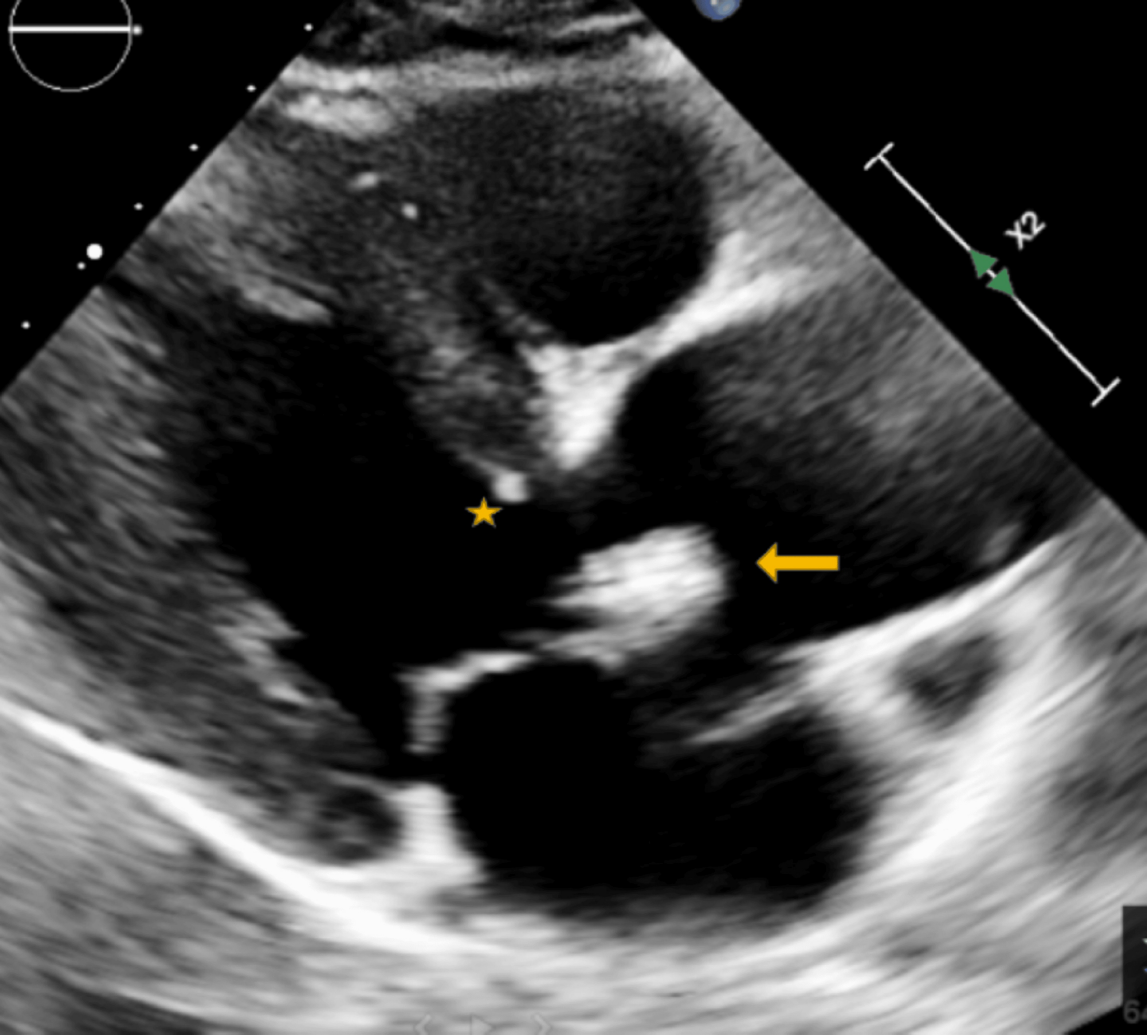
Parasternal long-axis TTE view demonstrating a discrete subaortic membrane (asterisk) located just below the aortic valve, along with focal calcification of the left coronary cusp (arrow). These findings contribute to subvalvular obstruction and structural valvular abnormalities noted on imaging X2 denotes a two-fold zoom applied by the echocardiography machine, indicating image magnification rather than an anatomical distance measurement. TTE: transthoracic echocardiogram

**Figure 4:**
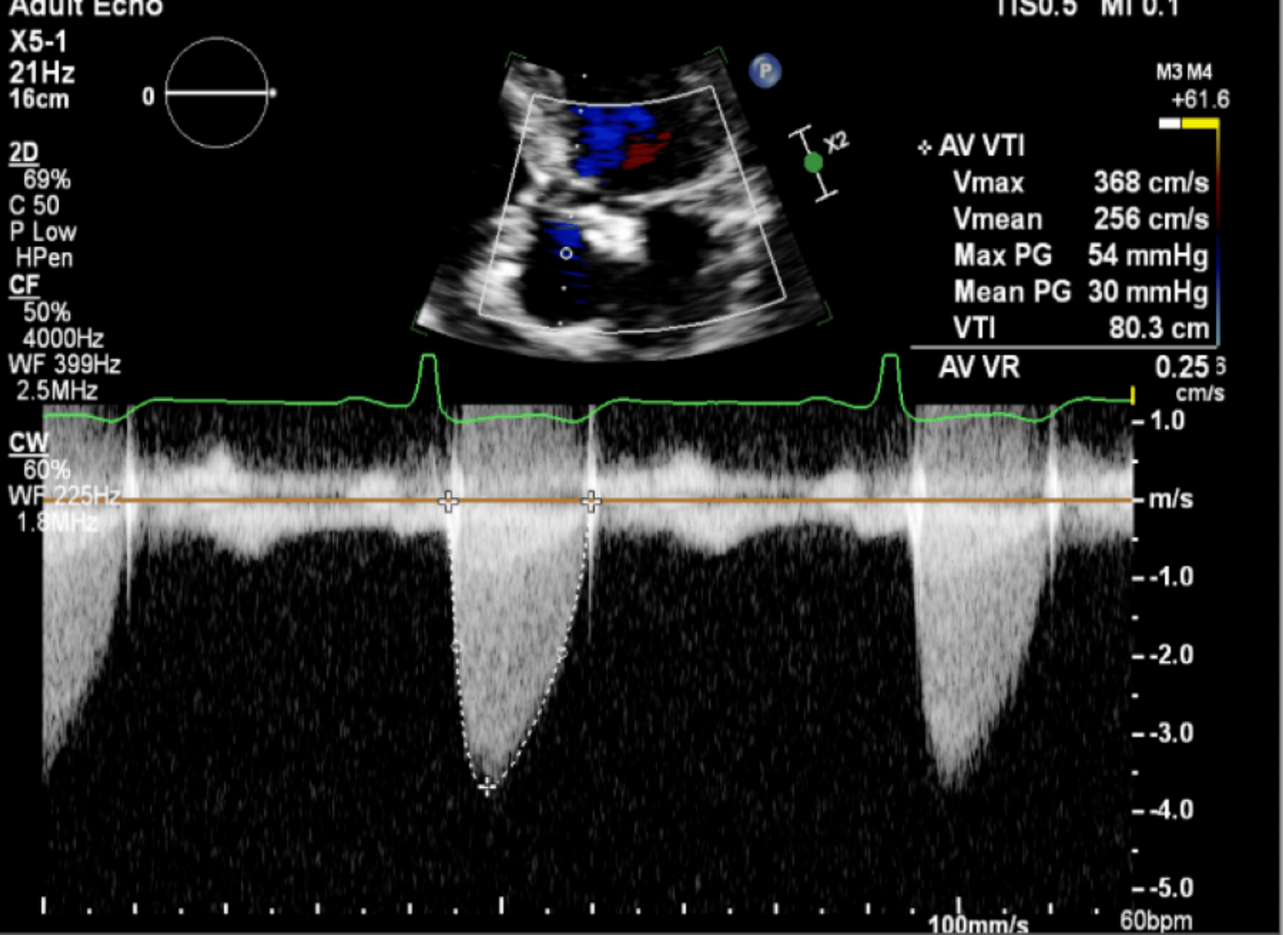
Continuous-wave Doppler interrogation across the aortic valve demonstrates an early-peaking systolic flow pattern with a mean transvalvular gradient of 30 mmHg, consistent with moderate aortic stenosis. The contour of the Doppler envelope raises concern for additional subvalvular obstruction, supporting suspicion of a subaortic membrane contributing to LVOT obstruction X2 denotes a two-fold zoom applied by the echocardiography machine, indicating image magnification rather than an anatomical distance measurement. LVOT: left ventricular outflow tract

**Figure 5:**
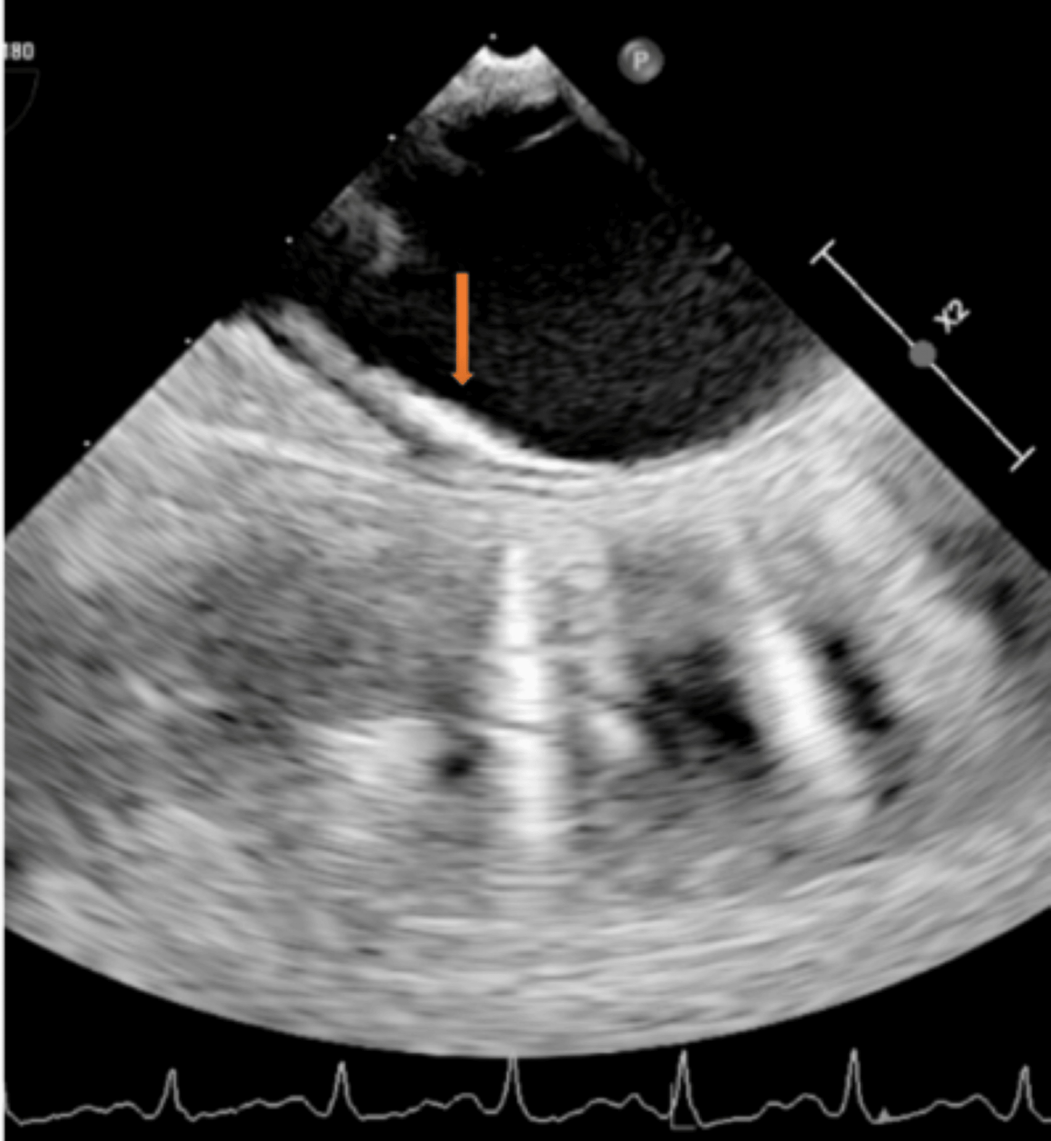
Atherosclerotic plaque in the aorta X2 denotes a two-fold zoom applied by the echocardiography machine, indicating image magnification rather than an anatomical distance measurement.

A TTE performed as part of the embolic workup showed preserved left ventricular systolic function, left atrial enlargement, and a probable BAV with thickened leaflets (Figure 1). Aortic root dilation was also noted (Figure 2), along with a subaortic membrane (Figure 3). Doppler examination suggested elevated LVOT gradients (Figure 4). No intracardiac thrombus was identified (Tables 2, 3). TTE also revealed atherosclerotic plaque in the ascending aorta (Figure 5).

**Table 2:** TTE summary: LV function, normal size, and systolic function (EF 55%) LVOT: left ventricular outlet tract; TTE: transthoracic echocardiography; LV: left ventricle; EF: ejection fraction

Anatomy of the heart	Comments
Left atrium	Dilated
Aortic valve	Thickened Leaflets and a bicuspid valve
LVOT	Elevated gradients; suspected subaortic membrane
Aorta	Mild ascending aortic dilation
Thrombus	None visualized

**Table 3:** TEE summary TEE: transthoracic echocardiogram

Anatomy	Comments
Aortic valve	Bicuspid (right-left cusp fusion), mild stenosis
Aortic root	Mild dilation (4.2 cm)
Aortic arch	Severe atherosclerotic plaque
Subaortic membrane	Not clearly visualized
Left atrium/left atrial appendage	No thrombus visualized
Bubble study	No intracardiac shunt

Given these findings, a TEE was obtained for further characterization. TEE confirmed a BAV with fusion of the right and left coronary cusps, mild aortic stenosis, and mild aortic root dilation measuring approximately 4.2 cm. Severe atherosclerotic plaque was visualized in the aortic arch, representing a plausible embolic source. No thrombus was identified in the left atrium or left atrial appendage, and an agitated saline study demonstrated no intracardiac shunt.

The patient was diagnosed with CRAO, likely secondary to embolization from severe aortic arch atherosclerosis in the setting of previously unrecognized structural heart disease. Fundoscopic examination revealed a pale macula with an area of wedge-shaped pallor surrounding the retinal vessels (Table 4). He was started on dual antiplatelet therapy, a high-intensity statin, and antihypertensive medications. Smoking cessation counseling was provided. He was discharged with close follow-up arranged with cardiology, cardiothoracic surgery for surveillance of aortic root dilation, neurology for evaluation of MRI abnormalities, and ophthalmology for ongoing visual assessment.

**Table 4:** Fundoscopic eye exam findings C/D ratio: cup-to-disc ratio, a key measurement used to assess the optic nerve head. It compares the size of the optic cup (the pale central depression) to the size of the optic disc (the entire circular nerve head).

Fundus exam	Right	Left
Disc	Normal	Normal
Macula	Normal	Pale macula with an area of wedge pallor around the vessels
C/D ration	0.7	0.7
Vessels	Tortuous	Tortuous
Periphery	Tigroid	Tigroid

## Discussion

This case illustrates a rare combination of BAV, aortic root dilation, and a subaortic membrane that was identified during the evaluation of an embolic retinal event. Each component of this triad carries distinct clinical implications, and their coexistence may reflect shared developmental pathways or complex hemodynamic interactions.

BAV is strongly associated with progressive dilation of the ascending aorta, a process thought to result from intrinsic abnormalities of the aortic wall as well as altered flow dynamics [1,2]. Patients with BAV therefore have a markedly increased risk of developing aortic aneurysm and aortic dissection over their lifetime [3]. Current guidelines recommend individualized surveillance strategies based on aortic dimensions, the rate of aortic enlargement, and relevant family history [4]. Despite these potential complications, two large contemporary series have demonstrated that overall life expectancy in adults with BAV disease is not reduced compared with that of the general population [1].

DSS, caused by a subaortic membrane, is uncommon in adults and may be either congenital or acquired [5]. In a prevalence study of 2057 patients with congenital heart disease, 6.5% were found to have DSS [5]. This lesion may coexist with BAV, potentially as a result of abnormal flow patterns that promote fibrous membrane formation in the LVOT [6,7]. Subaortic membranes can increase LVOT gradients, accelerate aortic valve degeneration, and increase embolic risk due to turbulent flow. Transesophageal echocardiography is often required for definitive diagnosis, as TTE may underestimate the extent or severity of the membrane [8].

BAV is strongly associated with abnormalities of the proximal thoracic aorta, particularly progressive dilatation that can lead to aneurysm or dissection, reflecting a shared developmental defect of the valve and aortic wall. Patients with BAV require careful evaluation of valve function, the aortic root, and the ascending aorta, as aortic enlargement is common and may continue to progress over time [9].

CRAO is most commonly caused by emboli originating from the carotid arteries or the heart. In this case, severe aortic arch atherosclerosis was considered the most likely embolic source. Nevertheless, the presence of BAV, aortic root dilation, and a subaortic membrane underscores the importance of comprehensive cardiac evaluation in patients presenting with embolic events in the absence of clear carotid pathology.

The coexistence of BAV, aortic root dilation, and a subaortic membrane is rarely reported in adults. This constellation expands the recognized spectrum of BAV-associated structural abnormalities, such as those seen in Shone complex, and highlights the importance of multimodality imaging to ensure accurate diagnosis and avoid missed or underestimated pathology.

## Conclusions

This case illustrates a rare combination of subaortic membrane, BAV, and aortic root dilation presenting with an embolic retinal event in adulthood. Although this anatomy may resemble other congenital obstructive syndromes, it does not meet the criteria for Shone complex, highlighting the wide spectrum of congenital heart disease. Careful imaging and multidisciplinary evaluation were key to diagnosis and management.
